# Bilateral Diffuse Uveal Melanocytic Proliferation Secondary to Rectal Adenocarcinoma: A Case Report and Literature Review

**DOI:** 10.3389/fmed.2021.691686

**Published:** 2021-07-20

**Authors:** Nianting Tong, Liangyu Wang, Nan Wang, Zhanyu Zhou

**Affiliations:** Department of Ophthalmology, Qingdao Municipal Hospital, Qingdao, China

**Keywords:** bilateral diffuse uveal melanocytic proliferation, case report, paraneoplastic syndrome, exudative retinal detach, rectal adenocarcinoma

## Abstract

**Background:** Bilateral diffuse uveal melanocytic proliferation (BDUMP) is a rare paraneoplastic intraocular syndrome that causes progressive visual loss in patients, and is associated with an underlying malignancy. Recently, the incidence of BDUMP has increased with the prolonged life expectancy of oncology patients.

**Case Presentation:** We report a case of a 68-year-old man with significant visual loss in both eyes. The patient presented with a diffusely thickened choroid and ciliary body, extremely shallow anterior chamber, increased intraocular pressure, and cataract formation, accompanied by exudative retinal detachment in both eyes. He underwent a pars plana vitrectomy and choroidal biopsy, which revealed benign proliferation of melanocytes. A small amount of subretinal fluid persisted, and uveal thickness persisted in the early postoperative period. During the 1-year follow-up assessment, he underwent rectal tumor resection, and was pathologically diagnosed with rectal adenocarcinoma. Six months after the rectal tumor resection, the subretinal fluid was completely absorbed and the retina had reattached. The thickness of both the ciliary body and choroid had significantly decreased.

**Conclusion:** This case report describes a rare paraneoplastic intraocular syndrome, BDUMP, which was associated with rectal adenocarcinoma. Treatment for the primary malignancy gradually improved the visual symptoms and signs.

## Introduction

Bilateral diffuse uveal melanocytic proliferation (BDUMP), first described by Machemer (1), is a rare paraneoplastic syndrome affecting the eyes. Although the incidence of BDUMP was previously extremely low, with about 1.15 cases per year from 1980 to 2000, the incidence increased to 4.4 cases per year around 2017 because of increased disease awareness and an increased life expectancy among oncology patients (2). Ocular findings in BDUMP patients include multiple red-orange patches at the level of the retinal pigment epithelium (RPE) with early hyperfluorescence according to fluorescein angiography (FA), diffuse thickening of the uveal tract, rapidly progressive cataract, and exudative retinal detachment (3–6). BDUMP is caused by a diffuse proliferation of benign melanocytes in the uvea, predominantly in the choroid, and is histopathologically unrelated to the primary non-ocular tumor (3).

BDUMP usually occurs in patients with systemic malignant disease, specifically, ovarian carcinoma in women (4, 7–10) and lung carcinoma in men (3, 4, 11–13). Occasionally, BDUMP is associated with gastric adenocarcinoma (14, 15), colonic adenocarcinoma (16), bladder cancer (5, 17, 18), pancreatic carcinoma (19), or primary vitreoretinal lymphoma (20, 21).

We herein report a case of BDUMP. To the best of our knowledge, this is the first report of BDUMP associated with rectal adenocarcinoma. We present the results of comprehensive ophthalmic analyses and a detailed pathological examination.

## Case Presentation

**Figure 1:**
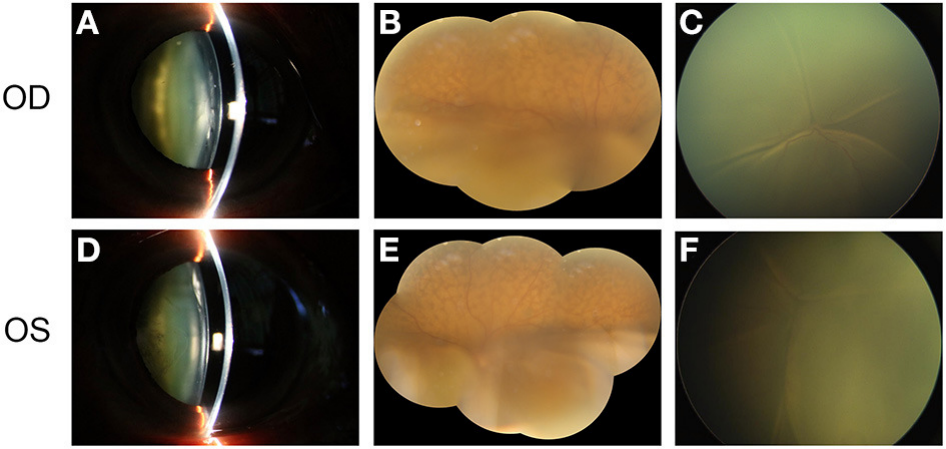
The main clinical manifestation of the patient before surgery. **(A)** shows the obvious cataract in the right eye of the patient. **(B)** shows the exudative retinal detachment in the inferior part of the retina, accompanied by naevus-like multifocal reddish patches in the superior part of the fundus. Total retinal detachment was observed in the posterior pole of the retina when the patient was in the supine position **(C)**. **(D–F)** show the similar clinical manifestation of the left eye.

**Figure 2:**
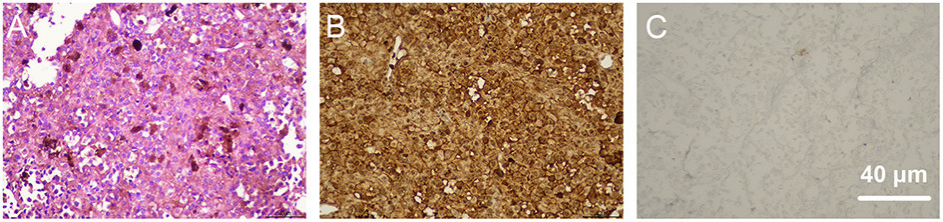
Pathological examination of biopsies collected from the choroid during surgery. **(A)** shows hematoxylin-eosin (HE) staining of the biopsies, which revealed a predominant spindle-shaped melanocytic cell mass. Immunohistochemical staining showed that the tissue was positive for antibodies against S-100 **(B)** and negative for antibodies against Ki67 **(C)**.

A 68-year-old Chinese man complained of gradual visual loss that occurred in both eyes over a period longer than 9 months. Approximately 6 months before he came to our clinic, he underwent a subtotal pneumonectomy to treat a pulmonary tumor, for which the pathological diagnosis was benign. Two months before he came to our clinic, assessments at another medical center revealed increased intraocular pressure (IOP) (around 30 mmHg in both eyes), a whole circle thickened ciliary body according to ultrasonic biomicroscopy (UBM), and decreased anterior chamber depth. He was able to count fingers in an assessment of best corrected visual acuity (BCVA) for both eyes. The IOP was 19 mmHg for the right eye and 20 mmHg for the left eye. A slit-lamp examination of the anterior segment of the eyes showed obviously tortuous and dilated episcleral vessels (Supplementary Figure 1), significantly decreased depth of anterior chamber, iris bombe, slight eversion of iris pigment at the pupillary margin and prominent cortical opacity of the lens (Figure 1). There were no signs of synechia, neovascular membranes in the iris, or active inflammation (Figure 1). Open angles with pigmentation within the trabecular meshwork were revealed by gonioscopy. Color fundus retinal photography revealed typical exudative retinal detachment in both eyes (Figure 1), accompanied by naevus-like multifocal reddish patches under RPE in the superior part of the fundus. Ocular coherence tomography (OCT) showed exudative retinal detachment of the macula with multiple hyperreflective elevated lesions under RPE. Lensectomy, peripheral iridectomy, diagnostic pars plana vitrectomy (PPV), and silicon oil tamponade were performed on the right eye to deepen the anterior chamber, restore the transparency of the refractive media, reattach the retina, and take a choroid sample for biopsy. Vitreous fluid and subretinal fluid were collected during the surgery, but immunological analyses revealed no specific positive results. Haematoxylin-eosin (HE) staining for biopsies from the choroid suggested predominantly spindle-shaped melanocytic cell masses, while immunohistochemistry showed that the samples were positive for antibodies against S-100, and negative for antibodies against Ki67, LCA, PAX-5, and SOX-10 (Figure 2). The histopathological examination showed that the choroid lesion was a benign proliferation of melanocytes.

**Figure 3:**
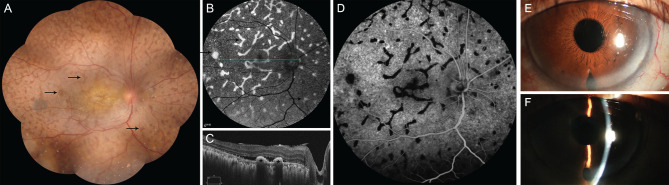
The main clinical manifestation of the patient after surgery. **(A)** shows a mass of giraffe-like multifocal reddish patches (arrow) in the retina. These patches were consistent with increased autofluorescence (**B**, arrow), decreased fluorescence in early phase of FFA **(D)**, and with multiple hyperreflective elevated lesions of the retinal pigment epithelium (**C**, asterisk), as revealed via ocular coherence tomography. It shows significantly deepening of the anterior chamber after surgery **(E,F)**.

One month after surgery in the right eye, the patient underwent surgery in the left eye. This had a similar procedure but no biopsy, and subretinal fluid was drained through the sclera. At a 1-week follow-up assessment, the BCVA of the patient had recovered to 20/100 for the right eye and 20/80 for the left eye, and the IOP was 21 mmHg for both eyes. The depth of the anterior chamber had increased and the iris bombe had disappeared (Figure 3). The retina was basically reattached with only a small amount of subretinal fluid. A mass of multifocal reddish patches (Figure 3) was found all around the retina, especially in the mid-peripheral sections. These patches were consistent with the elevated hyper-reflective mass under RPE (Figure 3) in the OCT data, and also with the hyperfluorescence in the autofluorescence images and hypofluorescence in the early FA data.

**Figure 4:**
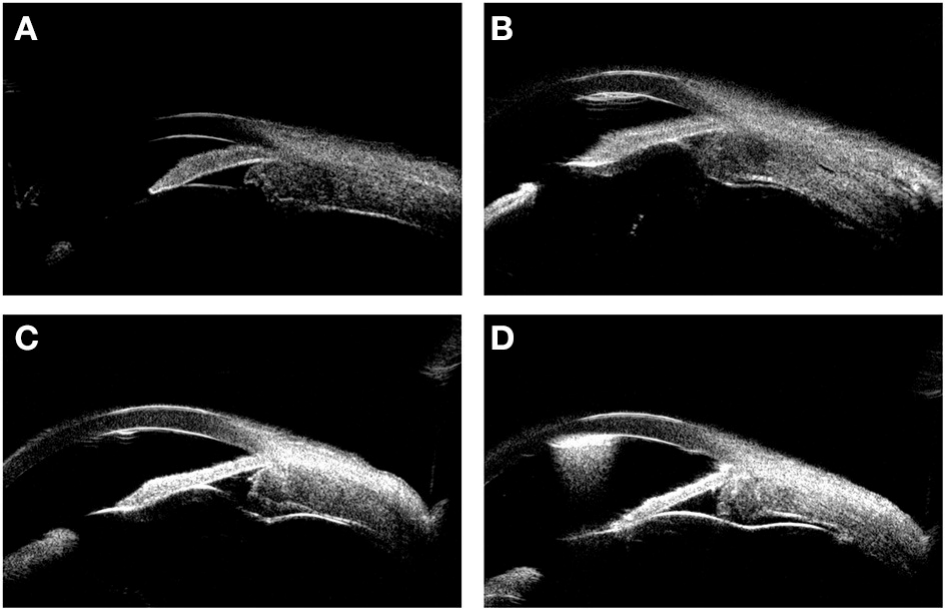
The UBM images showing the thickness variation during the surgery. **(A)** shows the thickened ciliary body and choroid before the surgery. **(B–D)** show the thickness variation of ciliary body and choroid 1, 9, 18 months (6 months after rectectomy) after the surgery, respectively.

A small amount of subretinal fluid persisted during the early postoperative follow-up period, and this was accompanied by thickening of the choroid. Three months after the operation, the patient underwent a rectectomy to treat a rectal tumor, and postoperative pathology revealed a rectal adenocarcinoma. Six months after the rectectomy, the subretinal fluid had completely disappeared and the thickness of the ciliary body and choroid had significantly decreased (Figure 4). The BCVA had improved to 20/60 for both eyes at the last follow-up.

## Discussion

### Diagnosis

Gass (6) first described the characteristics of BDUMP as multifocal red-orange subretinal patches with associated early hyperfluorescence in FA, scattered melanocytic tumors or thickening of the uveal tract, exudative retinal detachment, and rapid cataract progression. All of these features were present in our patient. As a type of paraneoplastic syndrome (22), BDUMP usually occurs in patients with systemic malignant disease, and the primary tumor is diagnosed after the presentation of BDUMP in nearly half of cases (3). In our case, the primary tumor was a rectal adenocarcinoma, and it was discovered more than 1 year after the appearance of BDUMP. No clear malignancy was found during treatment in the Department of Ophthalmology, and the patient was known only to have a history of subtotal pneumonectomy due to a pulmonary benign tumor. The diagnosis in this case was confusing to us, as the BDUMP diagnosis was made without any medical history of a primary malignancy.

### Histopathological Examination

According to the histopathological examination, biopsies from the pigmented intra-bulbar lesions showed predominantly spindle-shaped melanocytic cells with an occasional admixture of epithelial cells, but no or rare mitotic activity and no atypia (3). HE staining of biopsies collected from the choroid during surgery revealed a predominant spindle-shaped melanocytic cell mass, which was consistent with previous studies (9, 23). The immunohistochemical data for the biopsy provided useful information about the nature of the tissue. S-100 is normally present in cells derived from the neural crest (Schwann cells and melanocytes), chondrocytes, adipocytes, myoepithelial cells, macrophages, Langerhans cells, dendritic cells, and keratinocytes. S-100 can be found in melanomas, and, in some previous studies, was used as a biomarker for identifying melanocytes (24). In the present case, the positive expression of S-100 demonstrated that the tissue might have originated from melanocytes. Ki67 is a nuclear protein whose expression is strongly associated with tumor cell proliferation and growth (25, 26). Therefore, it is widely used in routine pathological investigations as a proliferation marker (26). In this case, the negative expression of Ki67 demonstrated the benign biological status of the tissue. When describing the characteristics of BDUMP, Gass (6) proposed that BDUMP be diagnosed differentially according to weather conditions resembled BDUMP before vs. after the development of multifocal pigmented choroidal tumors. BDUMP has been associated with B-cell lymphoma (20) and primary vitreoretinal lymphoma (21). Therefore, we conducted immunohistochemical staining against LCA, PAX-5, and SOX-10. The PAX-5 gene is a member of the paired box (PAX) family of transcription factors. PAX proteins are important regulators in early development, and alterations in the expression of their genes are thought to contribute to neoplastic transformation. The PAX-5 gene encodes B-cell lineage specific activator protein (BSAP) (27), which is expressed at early, but not late, stages of B-cell differentiation. As its expression has also been detected in the developing central nervous system, PAX-5 gene products may not only play an important role in B-cell differentiation (28), but also in neural development. The deregulation of PAX-5 transcription contributes to the pathogenesis of lymphomas and up to 97% of the Reed-Sternberg cells in patients with Hodgkin's lymphoma express Pax-5 (29). The SOX-10 gene encodes a member of the SOX (SRY-related HMG-box) family of transcription factors, which are involved in the regulation of embryonic development and determination of cell fate. Mutations in the SOX-10 gene have been associated with uveal melanoma (30). Because immunohistochemical staining against LCA, PAX-5, and SOX-10 was negative, we excluded the possibility of B-cell lymphoma and uveal melanoma.

### Cultured Melanocyte Elongation and Proliferation (CMEP) Factor

Although the exact pathogenesis of BDUMP and the associated mechanisms of uveal and dermal melanocytic proliferation remain unclear, CMEP factor from the IgG-rich fraction of serum from patients with BDUMP was previously found to stimulate melanocytic proliferation (31). Specifically, human melanocytes grew when they were exposed to serum or plasma from patients with BDUMP. Treatment of other cells, such as human dermal fibroblasts, keratinocytes, and ovarian cancer cells, with plasma from BDUMP patients indicated that the proliferation was melanocyte selective. This observation was supported by Jansen (32), who reported two cases of BDUMP in whom serum was collected. The serum from the first patient was subjected to plasmapheresis and did not demonstrate proliferation of cultured human melanocytes. However, the serum from the second patient was evaluated prior to treatment with plasmapheresis and did induce proliferation. In theory, CMEP factor should not have been present in the serum from the first patient but should have been present in the serum from the second patient. When human melanocytes were exposed to serum from the BDUMP patients, only the serum from the second patient induced melanocytic proliferation, confirming the ability of an IgG factor in BDUMP patients to induce proliferation.

### Treatment

Because systemic CMEP factor produced by systemic malignant diseases is considered to be involved in the pathogenesis of BDUMP, most previous case reports indicated that BDUMP treatment targeted the primary malignancies or metastases, including local resection, radiation, chemotherapy, or various combinations of these approaches (3, 14), and that visual symptoms and signs improved after the treatment. In our case, subretinal fluid was totally absorbed and the thickness of the ciliary body and choroid decreased with improvement in the BCVA score 6 months after the rectectomy. Plasmapherisis or plasma exchange is another way to remove CMEP in the plasma, and these approaches have effectively improved the BCVA and exudative retinal detachment in some BDUMP patients (12, 23, 32–36). In addition, anti-vascular endothelium growth factor agents and corticosteroids have been used to promote the absorption of subretinal fluid and improve the BCVA, although this treatment was not satisfactory (15, 35, 37).

### Prognosis

Although BDUMP is considered to be a benign proliferative disease, its prognosis is poor due to the primary malignancy, with an overall mean survival of 15.7 months after the presentation of BDUMP (3). Therefore, it is extremely important to establish diagnostic criteria enabling the timely detection of potential malignancies in patients with BDUMP.

## Conclusion

The typical clinical manifestations of BDUMP are multifocal red-orange subretinal patches, scattered melanocytic tumors or thickening of the uveal tract, exudative retinal detachment, and rapid cataract progression. Detailed systemic examinations to identify potential primary malignancies should be performed in patients with suspected BDUMP to facilitate early treatment and improve prognoses. In the present case, rectal adenocarcinoma was associated with BDUMP for the first time.

## Author Contributions

NT and LW made substantial contributions to the drafting of the article and conducted revisions of the manuscript. NW made substantial contributions to the acquisition, analysis, and interpretation of the data for this work. ZZ made substantial contributions to the conception and design of the work. All authors contributed to the article and approved the submitted version.

## Conflict of Interest

The authors declare that the research was conducted in the absence of any commercial or financial relationships that could be construed as a potential conflict of interest.

## Supplementary Material

Supplementary Figure 1 Photos from the anterior segment examination, which showed obviously tortuous and dilated episcleral vessels (**A–D** for the right eye showing the temporal, superior, inferior, and nasal side, respectively; **E–H** for the left eye showing the temporal, superior, inferior, and nasal side, respectively).
