# Supplementary figures and images for: Bilateral Diffuse Uveal Melanocytic Proliferation Secondary to Rectal Adenocarcinoma: A Case Report and Literature Review
Source: Front Med (Lausanne). 2021 Jul 20;8:691686. doi: 10.3389/fmed.2021.691686 (PMC8329363; doi:10.3389/fmed.2021.691686)

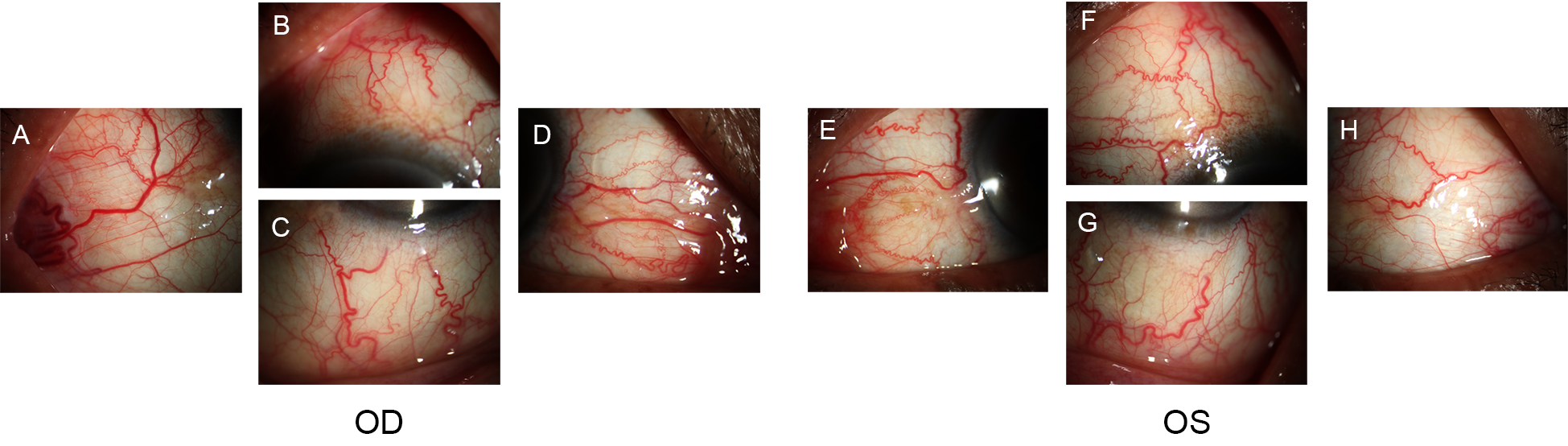

Supplement: Supplementary Figure 1 — Photos from the anterior segment examination, which showed obviously tortuous and dilated episcleral vessels (A–D for the right eye showing the temporal, superior, inferior, and nasal side, respectively; E–H for the left eye showing the temporal, superior, inferior, and nasal side, respectively). [file Image_1.tif]
